# Supplementary material for: Oral Trehalose Intake Modulates the Microbiota–Gut–Brain Axis and Is Neuroprotective in a Synucleinopathy Mouse Model
Source: Nutrients. 2024 Sep 30;16(19):3309. doi: 10.3390/nu16193309 (PMC11478413; doi:10.3390/nu16193309)
Supplement: Supplementary file 1 [file nutrients-16-03309-s001.zip › nutrients-3145945-supplementary.pdf]

nutrients

MDPI

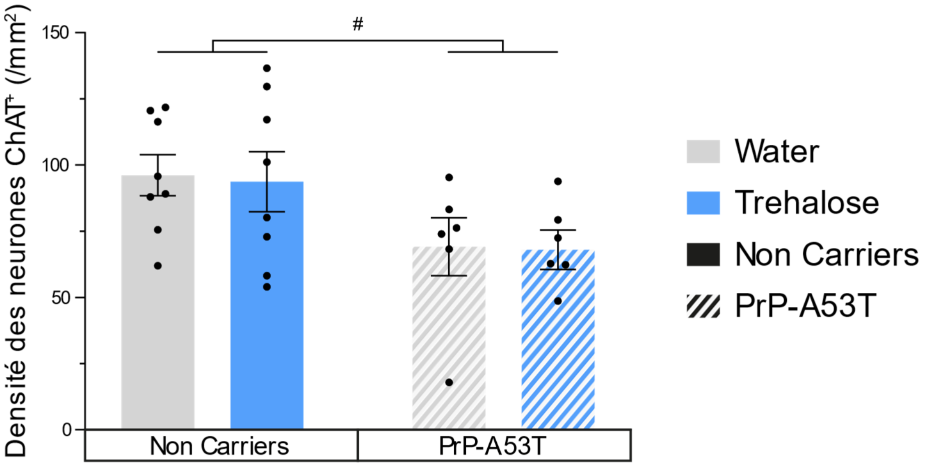

Figure S1. Effect of genotype and treatments on ChAT+ neurons density in the myenteric plexus of PrP-A53T and Non Carriers mice. Values shown are the mean density ± SEM of 6-8 mice per group. # $p < 0.05$ for the genotype effect (Non Carriers vs. PrP-A53T) 2-way ANOVA, Tukey test.

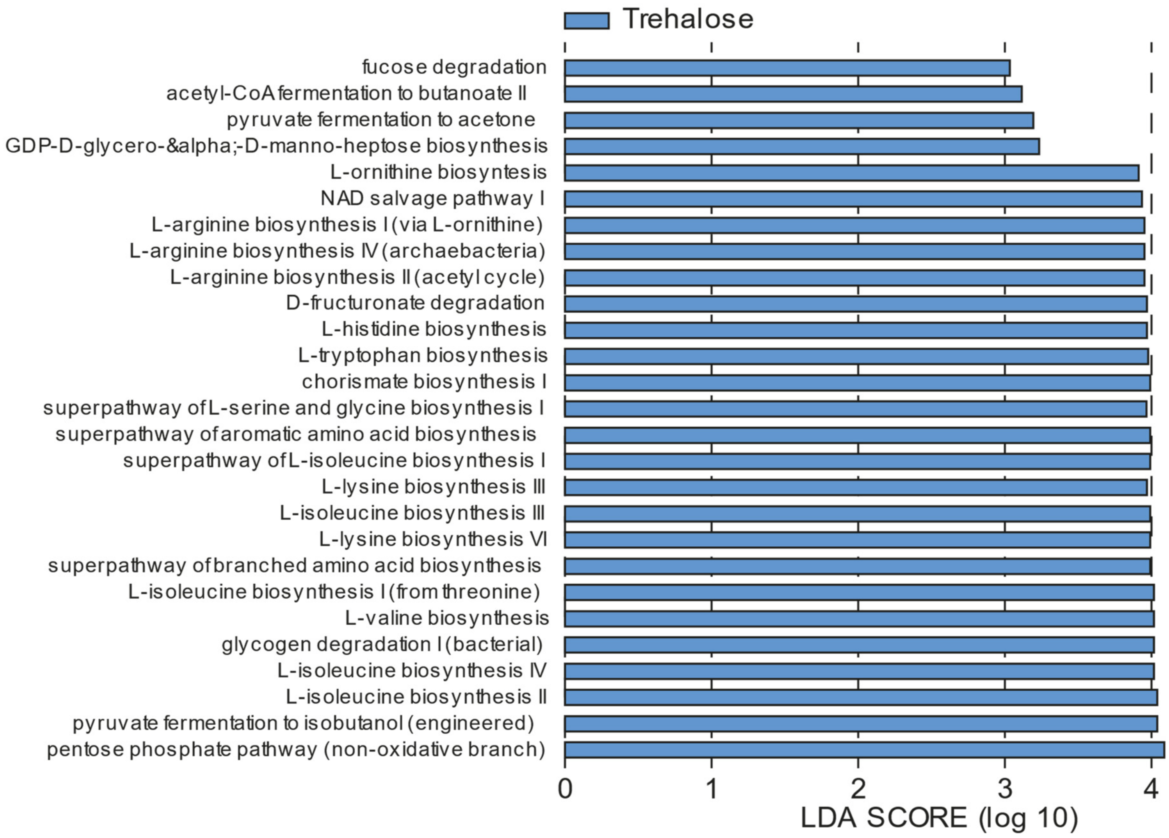

Figure S2. Relative abundance of predicted gut microbiota functional pathways with Linear Discriminant Analysis (LDA) scores among PrP-A53T mice treated with trehalose using LEfSe analysis. The LDA scores represent the effect size of each marker. Functional pathways in each group with an LDA score > 2 are considered.

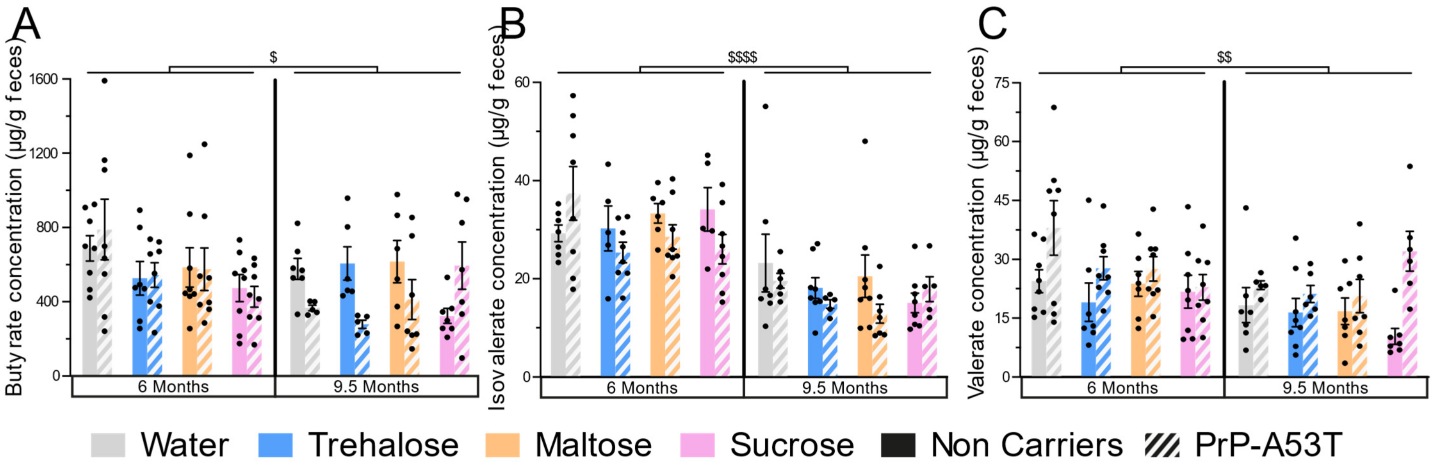

Figure S3. Effect of genotype and treatments on short chain fatty acids (SCFA) contents in feces. (**A**) Fecal butyrate contents at 6 months and 9.5 months of age. (**B**) Fecal isovalerate contents at 6 months and 9.5 months of age. (**C**) Fecal valerate contents at 6 months and 9.5 months of age. \$ $p < 0.05$, \$\$ $p < 0.01$, \$\$\$\$ $p < 0.0001$ for the age effect (6 Months vs. 9.5 Months); 3-way ANOVA, Tukey test. No significant differences were observed for the genotype effect (Non Carriers vs. PrP-A53T).
